# Reprogramming of DNA methylation and changes of gene expression in grafted *Hevea brasiliensis*

**DOI:** 10.3389/fpls.2024.1407700

**Published:** 2024-06-24

**Authors:** Hui-Liang Li, Ying Wang, Dong Guo, Jia-Hong Zhu, Yu Wang, Hao-Fu Dai, Shi-Qing Peng

**Affiliations:** ^1^ Key Laboratory of Biology and Genetic Resources of Tropical Crops, Institute of Tropical Bioscience and Biotechnology, Chinese Academy of Tropical Agricultural Sciences and Key Laboratory for Biology and Genetic Resources of Hainan Province, Hainan Academy of Tropical Agricultural Resource, Haikou, Hainan, China; ^2^ National Key Laboratory for Tropical Crop Breeding and Sanya Research Institute, Chinese Academy of Tropical Agricultural Sciences, Sanya, Hainan, China; ^3^ Hainan Key Laboratory for Biosafety Monitoring and Molecular Breeding in Off-Season Reproduction Regions, Institute of Tropical Bioscience and Biotechnology, Chinese Academy of Tropical Agricultural Sciences, Haikou, Hainan, China

**Keywords:** *Hevea brasiliensis*, grafting, whole genome bisulfite sequencing, DNA methylation, gene expression

## Abstract

Rubber tree (*Hevea brasiliensis*) is reproduced by bud grafting for commercial planting, but significant intraclonal variations exist in bud-grafted clones. DNA methylation changes related to grafting may be partly responsible for intraclonal variations. In the current study, whole-genome DNA methylation profiles of grafted rubber tree plants (GPs) and their donor plants (DPs) were evaluated by whole-genome bisulfite sequencing. Data showed that DNA methylation was downregulated and DNA methylations in CG, CHG, and CHH sequences were reprogrammed in GPs, suggesting that grafting induced the reprogramming of DNA methylation. A total of 5,939 differentially methylated genes (DMGs) were identified by comparing fractional methylation levels between GPs and DPs. Transcriptional analysis revealed that there were 9,798 differentially expressed genes (DEGs) in the DP and GP comparison. A total of 1,698 overlapping genes between DEGs and DMGs were identified. These overlapping genes were markedly enriched in the metabolic pathway and biosynthesis of secondary metabolites by Kyoto Encyclopedia of Genes and Genomes (KEGG) pathway analysis. Global DNA methylation and transcriptional analyses revealed that reprogramming of DNA methylation is correlated with gene expression in grafted rubber trees. The study provides a whole-genome methylome of rubber trees and an insight into the molecular mechanisms underlying the intraclonal variations existing in the commercial planting of grafted rubber trees.

## 1 Introduction

Rubber tree (*Hevea brasiliensis* Muell. Arg.) is the most unique commercial rubber-producing plant in the world. The propagation of rubber trees for commercial planting and conservation is being performed via artificial grafting scions (axillary buds) of elite clones onto rootstocks (unselected seedlings) to maintain the traits of interest (Hua et al., 2010). However, significant intraclonal variations exist in the growth and rubber yield of the bud-grafted clones, which is mainly attributed to the genetic heterogeneity of the stocks used for propagation (Chandrashekar et al., 1997; Zhou and Lin, 2012; Zhou et al., 2014). Although a great deal of effort has been invested in understanding the stock–scion interactions, most of the disparities reported were in terms of biochemical and phenotypic parameters (Huang and Lin, 2003; Ding et al., 2010; Zhou et al., 2014). The molecular mechanism of rootstock affecting scion growth and characteristics has not been fully understood in rubber trees.

DNA methylation, occurring in CG, CHG, and CHH contexts (where H represents C, T, or A), is one of the main epigenetic modifications. The establishment and maintenance of DNA methylation need several DNA methyltransferases, such as methyltransferase 1, DNA methyltransferase, and chromomethylases 2 and 3 (Law and Jacobsen, 2010; Zhang et al., 2018; Gallego-Bartolomé, 2020). The methylation of DNA plays an important role in plant cells, such as maintaining plant genome stability, chromosome interactions, gene expression, circRNA biogenesis, and mRNA stability and splicing (Law and Jacobsen, 2010; Zhang et al., 2018; Gallego-Bartolomé, 2020). DNA methylation affects plant physiology and development and regulates multiple biological processes, such as fertilization, floral pigmentation, floral scent, and photosynthesis as well as biotic or abiotic stress (Zhang et al., 2018; Gallego-Bartolomé, 2020; Mattei et al., 2022).

Grafting is a traditional method of plant asexual propagation by connecting scion and rootstock. In recent years, some studies have proposed the existence of DNA methylation changes in grafting reactions. The graft-induced DNA methylation in the scion has been connected with variations in grafted plants (Wu et al., 2013; Perrin et al., 2020; Santos et al., 2020; Cerruti et al., 2021; Kapazoglou et al., 2021; Han et al., 2022; Liu et al., 2023). Although grafting caused methylation variations in rubber trees, which were detected using methylation-sensitive amplified polymorphism (MSAP; Uthup et al., 2018), whole-genome DNA methylation by grafting in rubber trees remains largely unknown. Whole-genome bisulfite sequencing (WGBS) is a high-throughput and precise method for DNA methylation analysis, which can identify DNA methylation patterns genome-wide at single-base resolution (Smallwood et al., 2014; Li et al., 2018). Here, the genome-wide DNA methylation patterns between the grafted rubber tree plant (GP) and its donor plant (DP) were investigated using WGBS technology. Our findings reveal that grafting induced reprogramming of DNA methylation in GPs, with evidence suggesting a correlation between DNA methylation and gene expression. The study indicates that a potential molecular mechanism influences intraclonal variations existing in the commercial planting of grafted rubber trees.

## 2 Materials and methods

### 2.1 Plant material

Ten-year-old plants of tissue-cultured *H. brasiliensis* Muell. Arg. cultivar (CATA7–33-97) were planted in the National Rubber Tree Varieties Resource Garden of the Chinese Academy of Tropical Agriculture Sciences, Danzhou, Hainan, China. The seeds of the rubber trees (CATA7–33-97) were collected and germinated to produce seedlings, which were subsequently utilized as rootstocks after 1 year. The axillary buds of tissue-cultured rubber trees (CATA7–33-97) were taken and grafted onto the aforementioned rootstocks of the CATA7–33-97 variety. Mature leaf samples were collected from the 15 GPs when they grew to a stable phenology of the third canopy leaf and were pooled together to form one sample for DNA and RNA extraction. Concurrently, mature leaf samples were collected from the 15 DPs of tissue-cultured rubber trees (CATA7–33-97) and processed for DNA and RNA extraction to serve as a comparative control sample.

### 2.2 Whole-genome bisulfite sequencing

The isolation of genomic DNA was carried out as per the instruction of the Plant Genomic DNA Extraction Kit (TaKaRa, Dalian, China). Genomic DNA was checked on agarose gels. The purity and concentration of DNA were monitored using a NanoPhotometer^®^ spectrophotometer (IMPLEN, Westlake Village, CA, USA) and Qubit^®^ DNA Assay Kit in Qubit^®^ 2.0 Fluorometer (Life Technologies, Carlsbad, CA, USA).

For library construction, 200–300-bp DNA fragments were obtained by genomic DNA sonication, and then DNA fragments were combined with end-repair mix and adenylation. Cytosine-methylated adapters were ligated to the repaired DNAs, and subsequently, the connections were purified. The purified samples were treated with bisulfite as per the instruction of the Methylation Gold Kit (Zymo Research, Irvine, CA, USA). The treated DNA fragments were amplified to construct a DNA library. The library was qualified by the Qubit^®^ 2.0 Fluorometer (Life Technologies, CA, USA) and real-time PCR system (Bio-Rad, Hercules, CA, USA). The qualified libraries were sequenced on an Illumina NovaSeq platform (Novogene, Beijing, China).

### 2.3 Differentially methylated region analysis

The adaptor sequences in WGBS reads were removed using Trimmomatic (Bolger et al., 2014). The trimmed clean reads were aligned against the rubber tree reference genome (Liu et al., 2020) using Bismark (Krueger and Andrews, 2011). Methylated cytosines were extracted from aligned reads using the Bismark methylation extractor with default parameters. Considering inefficiencies in the bisulfite conversion reaction and sequencing errors, a binomial test was used to determine whether the observed methylation frequency was above the background [false discovery rate (FDR) <0.05]. The methylation level was assessed according to the previously described method (Lister et al., 2009). The different DNA methylation profiles in different genomic regions were identified according to Lister et al. (2013). Differentially methylated regions (DMRs) between GPs and DPs were determined using the R-package DSS-single (Feng et al., 2014; Wu et al., 2015). The CallDMR function was used to identify DMRs with parameters of twofold change and *p* ≤ 0.05 in methylation levels.

### 2.4 Transcriptome sequencing

Total RNA was extracted according to Tang et al. (2010). Total RNA purity was checked using the NanoPhotometer^®^ spectrophotometer (IMPLEN, CA, USA). Total RNA integrity was assessed using the RNA Nano 6000 Assay Kit of the Bioanalyzer 2100 system (Agilent Technologies, Santa Clara, CA, USA). Paired-end Illumina cDNA Sequencing libraries were generated following the manufacturer’s instructions for mRNASeq sample preparation (NEB, Ipswich, MA, USA). The library quality was assessed using the Agilent Bioanalyzer 2100 system (Agilent Technologies, CA, USA). The libraries were deep sequenced using the Illumina NovaSeq platform (Novogene, Beijing, China), and 150-bp paired-end reads were generated. Raw data (raw reads) of fastq format were first processed through in-house perl scripts. Clean data (clean reads) were obtained by removing reads containing adapter, reads containing ploy-N, and low-quality reads from raw data. At the same time, Q20, Q30, and GC content of the clean data were calculated. The clean reads were aligned against the rubber tree reference genome (Liu et al., 2020) using HISAT2 v2.0.5 (Kim et al., 2019), then transcripts were assembled, and gene expression levels were calculated using StringTie v1.3.3 (Pertea et al., 2016). The transcript abundance was calculated based on the ratio of fragments per kilobase of transcript per million mapped reads (FPKM) values, and the FDR (control method for the p-value threshold in multiple tests) was used for testing the significance of the differences. DESeq2 R package (v1.16.1) was used to identify differentially expressed genes (DEGs) with cutoff values of FDR ≤ 0.05 and |fold change| ≥ 2 (Love et al., 2014).

### 2.5 Integrated analysis of DNA methylation and the transcriptome

Pearson’s correlation analysis of DNA methylation and gene expression in DPs and GPs was completed as previously described (Yang et al., 2014). DMRs (<5% false discovery rate) are associated with significant changes in gene expression and enriched for an expected inverse relationship between methylation and expression (p < 2.2 × 10^−16^).

### 2.6 Functional enrichment analysis of overlapping genes between DEGs and DMGs

Gene Ontology (GO) enrichment analysis of overlapping genes between DEGs and differentially methylated genes (DMGs) was carried out using the GOseq R package at *p* ≤ 0.05 (Young et al., 2010). The statistical enrichment of overlapping genes between DEGs and DMGs in Kyoto Encyclopedia of Genes and Genomes (KEGG) pathways was performed using KOBAS software with Fisher’s exact test selecting *p* ≤ 0.05 (Mao et al., 2005).

### 2.7 Quantitative PCR

The total RNA of rubber tree leaves was extracted according to Tang et al. (2010). Quantitative PCR (qPCR) was performed using *HbACT7* as an internal reference gene (Li et al., 2014) according to the instruction of SYBR^®^Premix Ex Taq™ II Kit (TaKaRa, Dalian, China). The sequence of primers used in this study is listed in **Supplementary Table S1**. qPCR conditions were as follows: 95°C for 3 min; 35 cycles of 95°C for 5 s, 60°C for 30 s, 72°C for 20 s. The gene expression level was calculated using the 2(−Delta Delta C (T)) method (Livak and Schmittgen, 2001).

## 3 Results

### 3.1 Grafting induced DNA methylation change in *H. brasiliensis*

**Figure 1:**
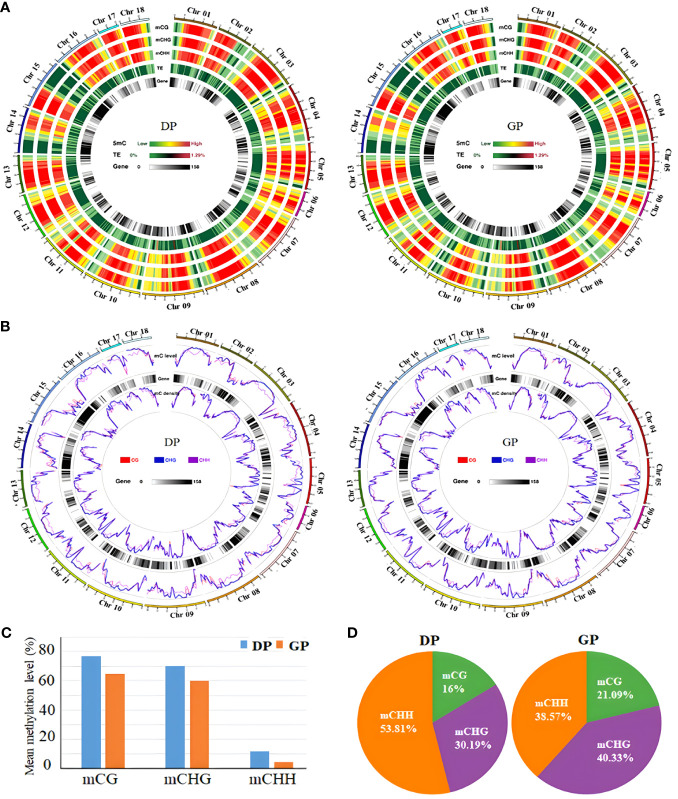
Grafting induced DNA methylation change in *Hevea brasiliensis*. **(A)** Chromosome-level methylation features in DPs and GPs. Track from the outside to the inside, as follows: mCG, mCHG, mCHH, TEs, and gene. **(B)** Density plot of 5mC in three contexts in the gene bodies on each chromosome in DPs and GPs. **(C)** The mean methylation levels in three contexts between DPs and GPs. **(D)** The levels of mC in CG, CHG, and CHH in DPs and GPs. DPs, donor plants; GPs, grafted rubber tree plants; TEs, transposable elements.

To clarify the possible effects of rubber trees with grafting on DNA methylation levels, WGBS was performed to investigate whole-genome methylation in GPs and DPs. In total, 204,849,774 clean reads (56.64 G) and 220,533,308 clean reads (60.71 G) were obtained from GPs and DPs. More than 99.23% of DPs and 99.55% of GPs of cytosines were converted by bisulfite treatment, which showed that the conversion was satisfactory with WGBS. In total, 123,873,186 clean reads in DPs and 119,464,270 clean reads in GPs were mapped to the rubber tree reference genome (**Table 1**). The genome-wide methylation for chromosomes and distinct genomic elements were characterized in DPs and GPs (**Figures 1A, B**). The proportions of methylated cytosines (mC) decreased from 26.2% in DPs to 16.48% in GPs (**Table 1**). The mean methylation levels decreased from 77% in DPs to 64.86% in GPs in the CG context, from 72.02% in DPs to 59.83% in GPs in the CHG, and from 11.8% in DPs to 4.36% in GPs in the CHH (**Figure 1C**). Relative proportions of mC in the CG, CHG, and CHH contexts were respectively 16%, 30.19%, and 53.81% in DPs. In GPs, 21.09%, 40.33%, and 38.75% of mC in CG, CHG, and CHH, respectively, were detected (**Figure 1D**). CHH methylation significantly decreased from 53.81% in DPs to 38.75% in GPs, while CG and CHG methylation increased from 16% and 30.19% in DPs to 21.09% and 40.33% in GPs, respectively. The above data suggested that grafting induced reprogramming of DNA methylation in grafted rubber trees.

**Table 1:** Data summary of whole-genome bisulfite sequencing for the two rubber tree samples.

Sample	Clean reads	Clean bases	BS conversion rate	Mapped reads	Mean C mC/C (%)	Mean CG mCG/CG (%)	Mean CHG mCHG/CHG (%)	Mean CHH mCHH/CHH (%)	mCG percent (%) mCG/mC	mCHG percent (%) mCHG/mC	mCHH percent (%) mCHH/mC
DP	204,849,774	56.64 G	99.23%	123,873,186	26.2%	77%	72.03%	11.8%	16%	30.19%	53.81%
GP	220,533,308	60.71 G	99.55%	119,464,270	16.48%	64.86%	59.83%	4.36%	21.09%	40.33%	38.57%

DP, donor plant; GP, grafted rubber tree plant.

### 3.2 Grafting changed methylation levels in gene regions

**Figure 2:**
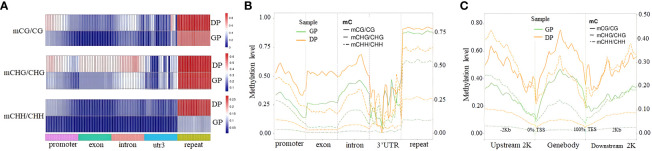
DNA methylation profiles in different elements of genes. **(A)** Heat map of methylation levels in three contexts of different elements of genes. **(B)** Distribution of DNA methylation levels in three contexts among elements of genes. **(C)** Methylation levels in three contexts in 2-kb upstream region, gene body, and downstream region in DPs and GPs. DPs, donor plants; GPs, grafted rubber tree plants.

The analysis of DNA methylation levels in different gene regions indicated that DNA methylation levels in various gene regions had significant differences between GPs and DPs (**Figure 2A**). The DNA methylation levels in three contexts of gene regions except for 3′UTR in DPs were more than those in GPs (**Figure 2B**). Meanwhile, DNA methylation levels in all contexts of gene-body regions, upstream 2K, and downstream 2K were lower in the GPs than in the DPs (**Figure 2C**). Taken together, grafting changed methylation levels in gene regions.

### 3.3 Analysis of DMGs between GPs and DPs

**Figure 3:**
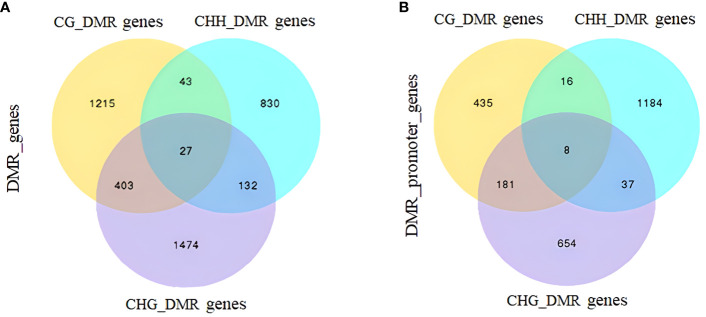
Venn analysis of DMGs between DPs and GPs. **(A)** Gene-body regions (DMR_genes). **(B)** The promoter regions (DMR_promoter_genes). DMGs, differentially methylated genes; DPs, donor plants; GPs, grafted rubber tree plants.

**Figure 4:**
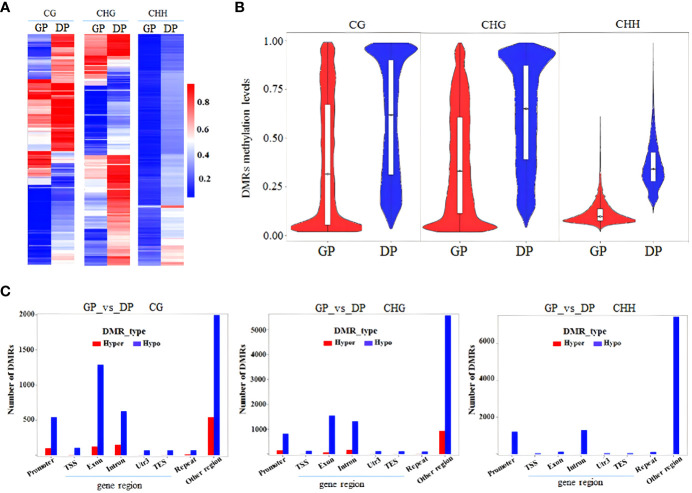
Comparative analysis of DMGs between DPs and GPs. **(A)** Heat map of methylation of DMGs in three contexts. **(B)** DMR methylation levels in three contexts. **(C)** Number of DMRs (hyper-/hypomethylated) in three contexts in the different gene regions. DMGs, differentially methylated genes; DPs, donor plants; GPs, grafted rubber tree plants; DMR, differentially methylated region.

**Figure 5:**
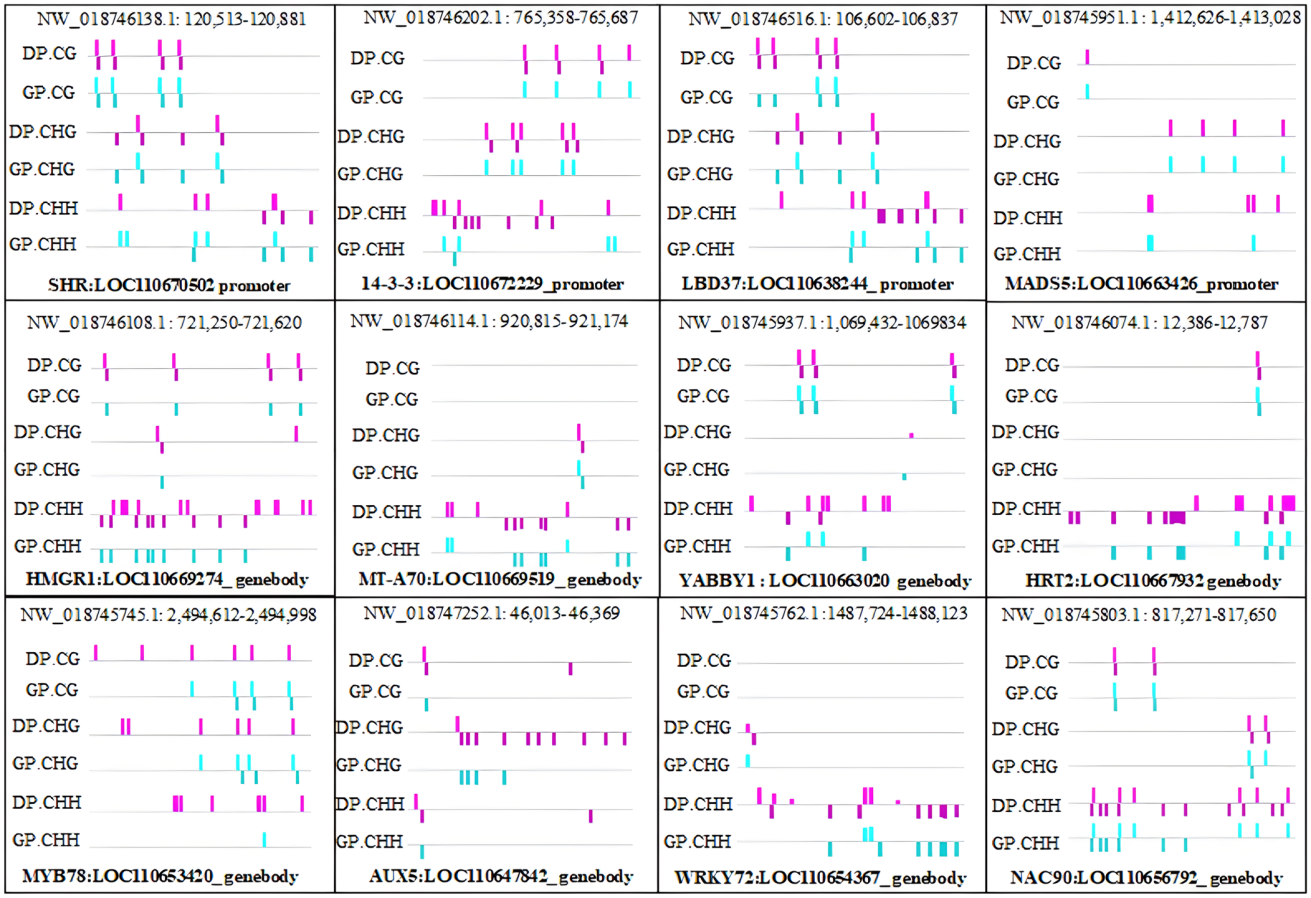
IGV snapshots of the representative DMGs in DPs and GPs. DMGs, differentially methylated genes; DPs, donor plants; GPs, grafted rubber tree plants.

DMRs and DMGs were identified by comparing fractional methylation levels between GPs and DPs. A total of 7,570 (819 hyper- and 6,751 hypomethylated) DMRs were identified. According to the location of DMRs, DMGs were categorized into DMR-associated promoter genes and DMR-associated genes. A total of 4,894 DMR-associated genes and 2,676 DMR-associated promoter genes were observed between GPs and DPs (**Figures 3A, B**). For mCG, 2,135 DMR-associated genes and 596 DMR-associated promoter genes were obtained in the comparison. For mCHG, there were 2,129 DMR-associated genes and 719 DMR-associated promoter genes between GPs and DPs. For mCHH, there were 1,487 DMR-associated genes and 1,548 DMR-associated promoter genes between GPs and DPs, respectively. In DMR-associated genes, the number of DMGs in mCG was the highest in all contexts, and the number of DMGs in mCHH was the lowest in all contexts. In DMR-associated promoter genes, the number of DMGs in mCHH was the highest in all contexts, and the number of DMGs in mCG was the lowest in all contexts. A heat map was also used to visualize the DMGs and their DNA methylation levels in three contexts between GPs and DPs (**Figure 4A**). Additionally, the methylation levels in the three contexts of the DMGs were obviously lower in the GPs than in the DPs (**Figure 4B**). The DMR distribution in three contexts in the genic regions varied in GPs and DPs. For the CG and CHG contexts, there were considerably more hypomethylated DMRs than hypermethylated DMRs in all gene regions in GPs and DPs. For the CHH context, there were few hypomethylated DMRs in all gene regions in GPs and DPs (**Figure 4C**). Thus, the DMR distribution and DNA methylation levels in genic regions may be regulated by grafting. Moreover, the IGV snapshots of the DMGs in GPs and DPs are presented in **Figure 5**. These results suggested that there were many differentially methylated genes between GPs and DPs.

### 3.4 Grafting induced changes of gene expression in GPs

**Figure 6:**
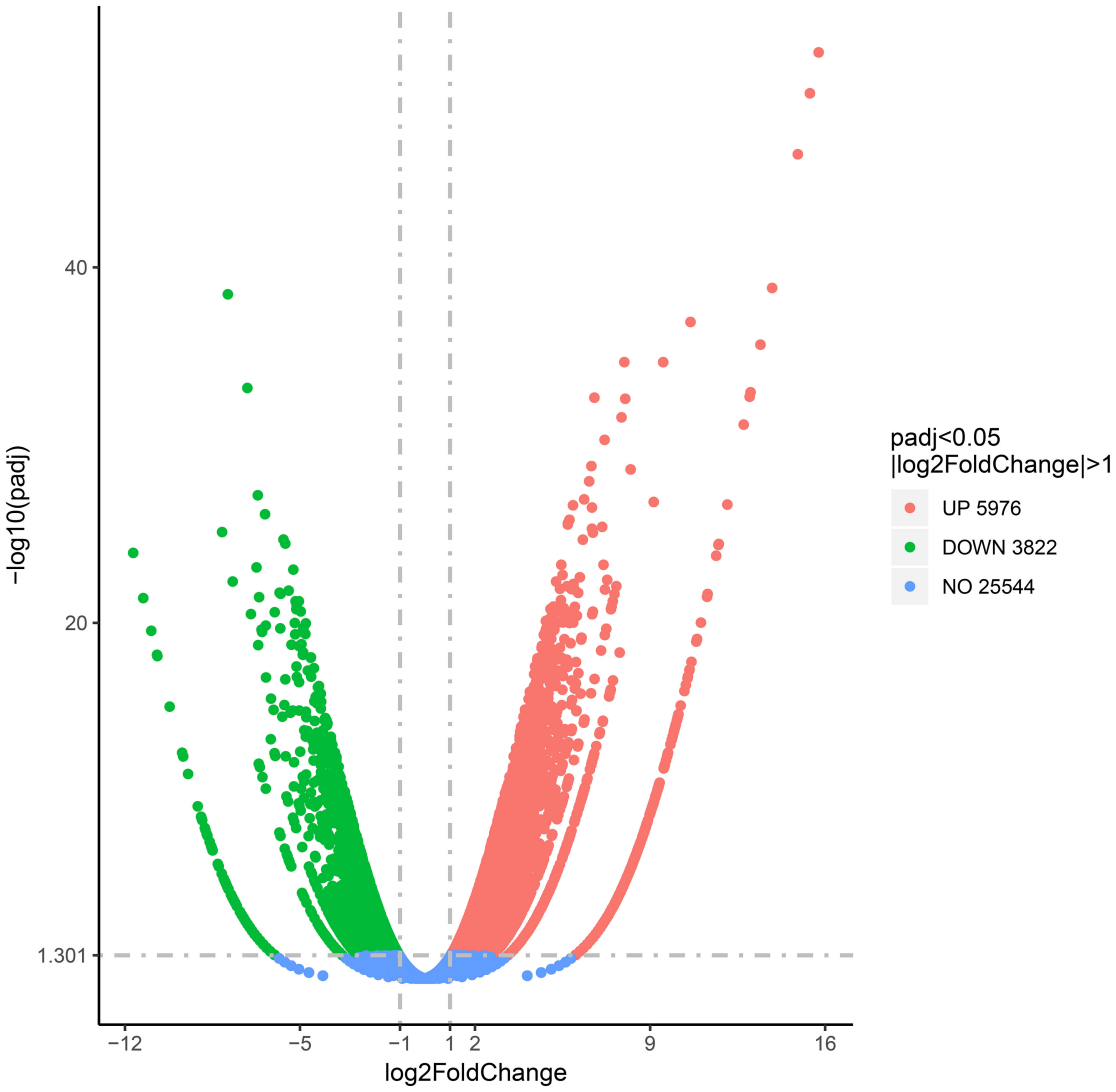
Volcano plot of differentially expressed genes (DEGs) in the DP and GP comparison. DP, donor plant; GP, grafted rubber tree plant.

To study the correlation of gene expression and overlapping DMGs, the transcriptome analysis of GPs and DPs was accomplished. In total, 45,078,466 clean reads (6.76 G) and 44,625,118 clean reads (6.69 G) were acquired from DPs and GPs (**Supplementary Table S2**), respectively. A total of 9,798 DEGs were identified in the DP and GP comparison. Among DEGs, 5,976 DEGs were upregulated and 3,822 DEGs were downregulated (**Figure 6**, **Supplementary Table S3**).

### 3.5 DMRs correlated with gene expression levels in GPs and DPs

**Figure 7:**
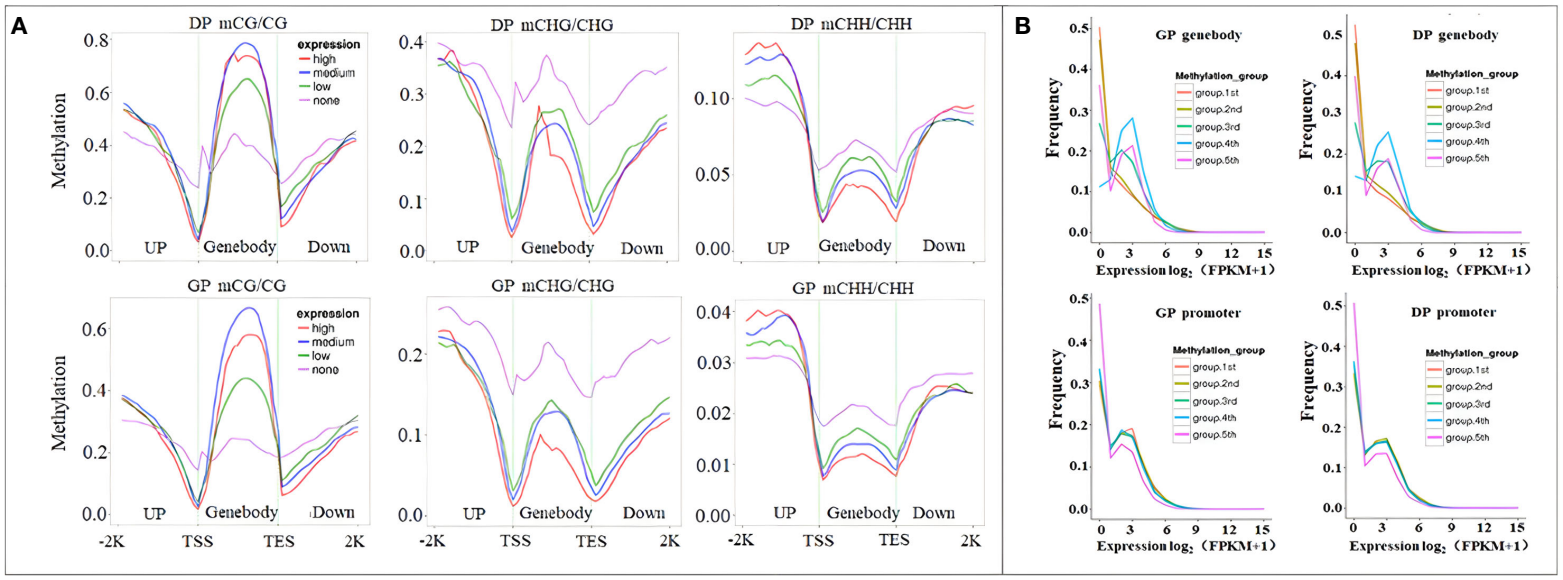
Correlation between DNA methylation and gene expression. **(A)** Distribution of methylation levels within gene bodies partitioned by different expression levels. **(B)** Comparison of the expression profiles of methylated and unmethylated genes.

To investigate the relationship between methylation patterns and gene expression levels in rubber tree, genes were classified into the following quartiles based on their FPKM values: no expression (FPKM < 1), low expression (1 < FPKM < the lower quartile), medium expression (the lower quartile < FPKM < the upper quartile), and high expression (FPKM > the upper quartile). Highly expressed genes exhibited lower methylation levels in the CHG and CHH contexts in the gene-body and downstream regions but higher methylation levels in the CHH context within the upstream regions. The non-expressed genes displayed high methylation in CHG within the gene-body, upstream, and downstream regions and low methylation in the CG context within the gene-body regions. Furthermore, non-expressed genes showed lower methylation in CG in the gene-body regions but high methylation in CHG in the gene-body regions. Additionally, the non-expressed genes were highly methylated in all three contexts in downstream regions. In contrast, medium-expressed genes showed high methylation levels in the CG context in upstream regions and gene-body regions (**Figure 7A**).

Meanwhile, based on the methylation level in the promoter and gene-body region, methylated genes were classified into five groups: group 1 (methylation level < 20%), group 2 (20%–40%), group 3 (40%–60%), group 4 (60%–80%), and group 5 (>80%). Genes in group 5 within the gene-body region, with the lowest methylation levels, exhibited the lowest expression levels, while genes in group 1 with the lowest methylation level in the promoter region showed the lowest expression levels (**Figure 7B**), indicating a correlation between methylation status and gene expression levels.

**Figure 8:**
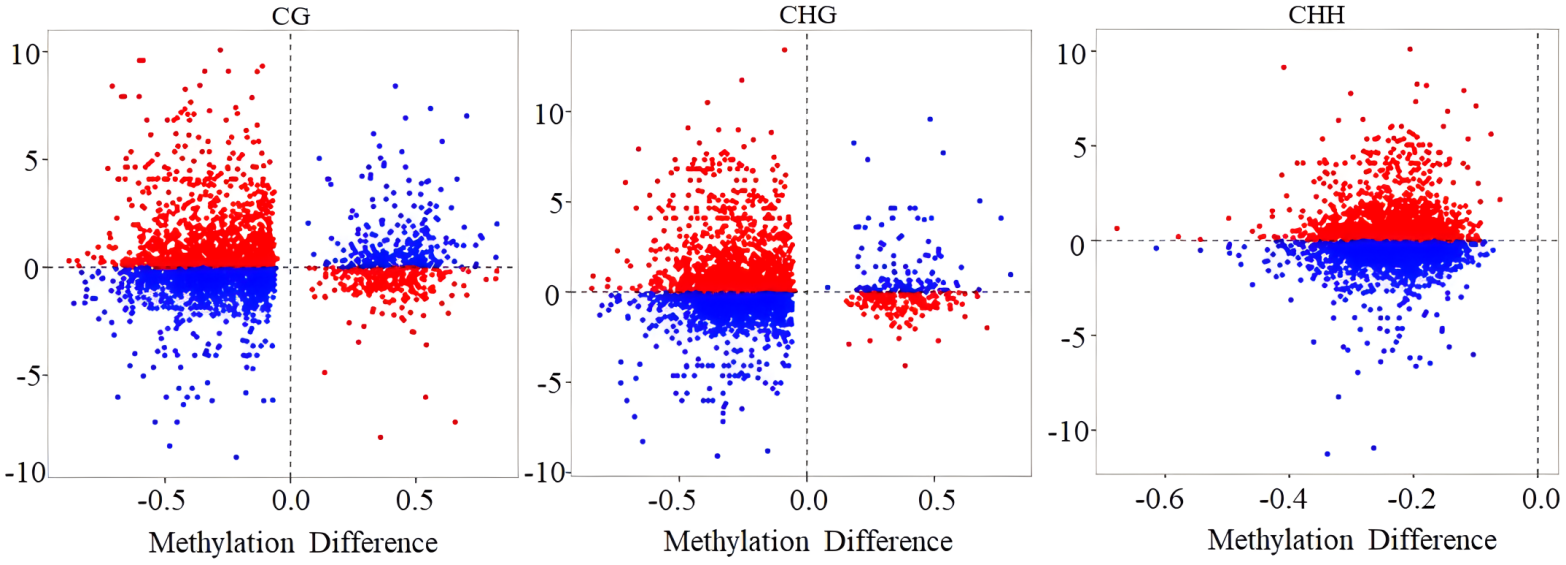
The scatter plot of the relationship between methylation level and gene expression level. Red and blue dots represent the gene–differentially methylated region (DMR) pairs exhibiting either inverse or equivalent relationships, respectively.

To determine the correlation between gene methylation and gene expression levels, a scatter plot was employed to visualize the methylation level of DMRs along with their corresponding transcriptome differential gene expression levels. The results showed a positive correlation between hypomethylated DMRs and elevated gene expression, and hypermethylated DMRs correlated with decreased gene expression (red dots in **Figure 8**). However, in the CG, CHG, and CHH contexts, 47.87%, 49.18%, and 54.18% methylation changes did not demonstrate the anticipated associations with gene expression changes, respectively (blue dots in **Figure 8**).

### 3.6 Functional enrichment analysis of overlapping genes between DEGs and DMGs

**Figure 9:**
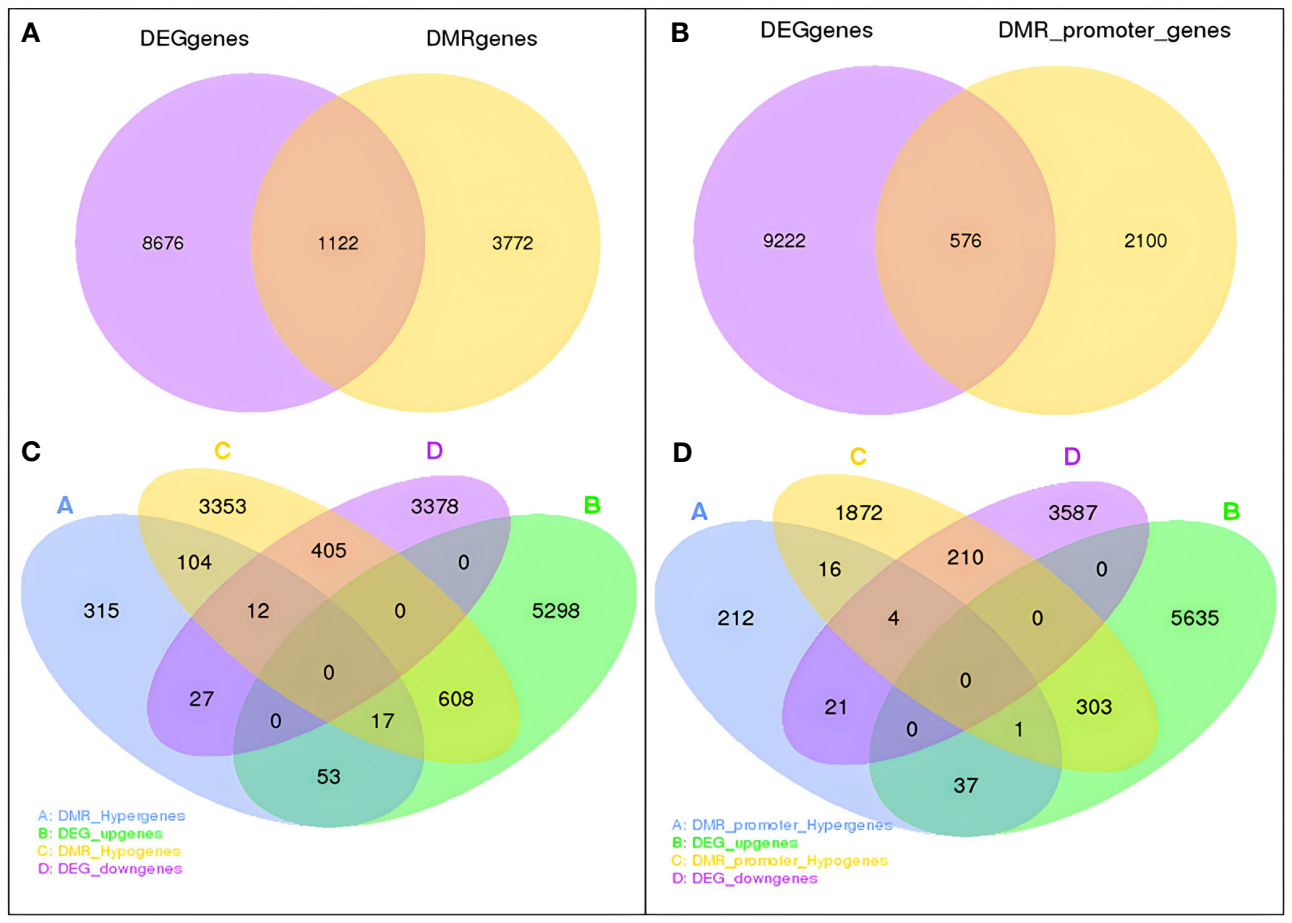
Association of DMRs with DEGs in the DP vs. GP comparisons. **(A)** Venn analysis of overlapping genes between DMR_genes and DEGs. **(B)** Number of overlapping genes between DMR_promoter genes and DEGs. **(C)** Venn analysis of upregulated and downregulated DEGs overlapped with hypermethylated and hypomethylated DMR_genes. **(D)** Venn analysis of upregulated and downregulated DEGs overlapped with hypermethylated and hypomethylated DMR_promoter genes. DMRs, differentially methylated regions; DEGs, differentially expressed genes; DP, donor plant; GP, grafted rubber tree plant.

Among these obtained DEGs and DMGs, 1,698 overlapping genes between DEGs and DMGs were identified. In detail, 1,122 genes overlapped with DMR_genes, and 576 overlapped with DMR_promoter_genes (**Figures 9A, B**). A total of 39 and 417 downregulated DEGs overlapped with hypermethylated and hypomethylated DMR_genes, while 70 and 625 upregulated DEGs overlapped with hypermethylated and hypomethylated DMR_genes. In addition, 68 and 304 upregulated DEGs overlapped with hypermethylated and hypomethylated DMR_promoter genes, and 25 and 214 downregulated DEGs overlapped with hypermethylated and hypomethylated DMR_promoter genes (**Figures 9C, D**).

**Figure 10:**
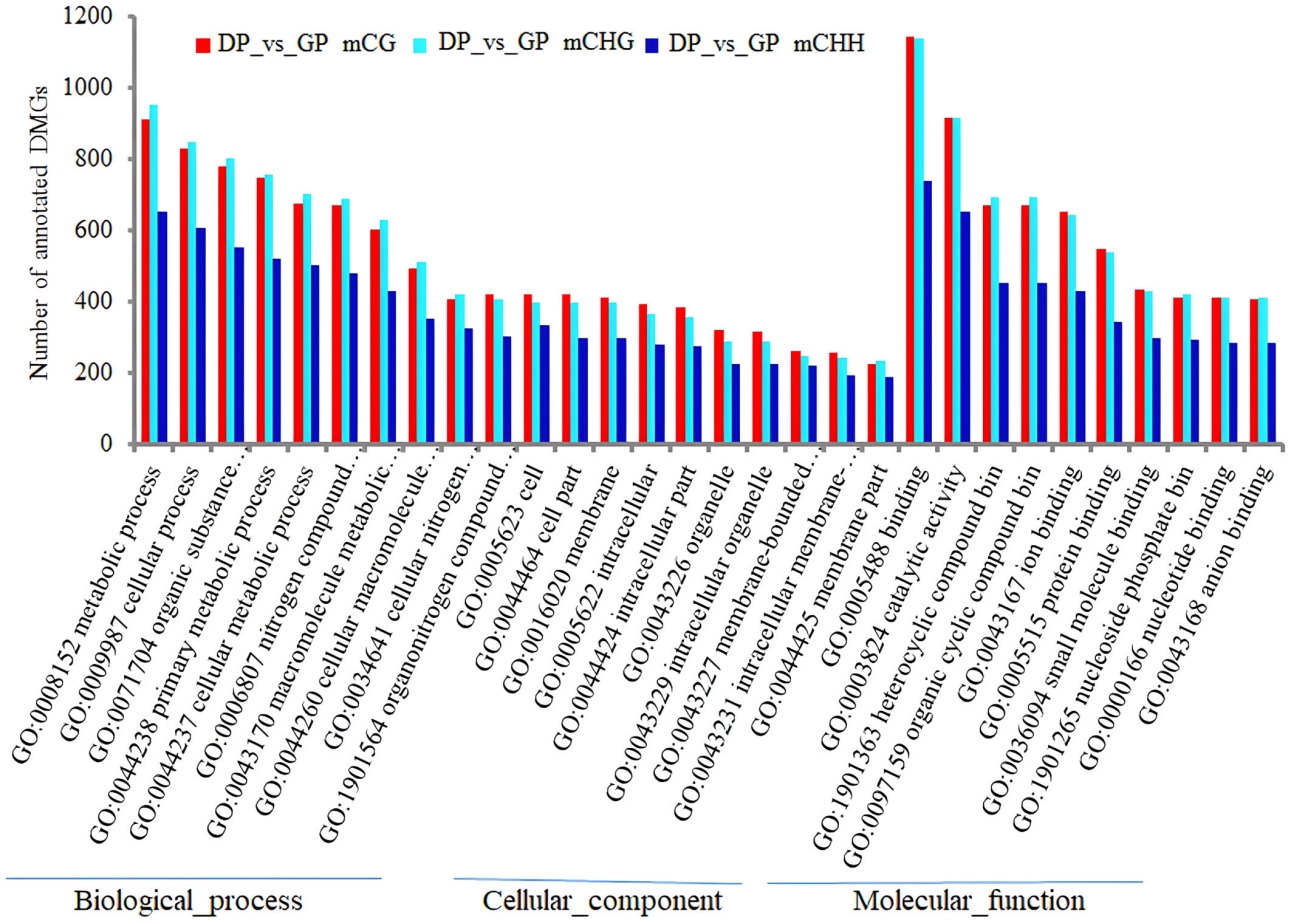
The enrichment analysis of GO terms (top 10) of DMGs in three contexts between DPs and GPs. GO, Gene Ontology; DMGs, differentially methylated genes; DPs, donor plants; GPs, grafted rubber tree plants.

The functional enrichment analysis of overlapping genes in three contexts was carried out by GO and KEGG analyses. The analysis of GO annotation enrichment is shown in **Figure 6**. The DMGs in CHH were significantly less in the term of cellular component (CC) than in biological process (BP) and molecular function (MF) than in CG and CHG. In the term of BP, the DMGs were mostly joined in the metabolic process, followed by the cellular process, organic substance metabolic process, primary metabolic process, and cellular metabolic process. In the term of MF, DMGs mostly participated in binding, catalytic activity, heterocyclic compound binding, organic cyclic compound binding, and ion binding. In the term of CC, DMGs were mainly correlated with cell, cell part, and membrane (**Figure 10**).

**Figure 11:**
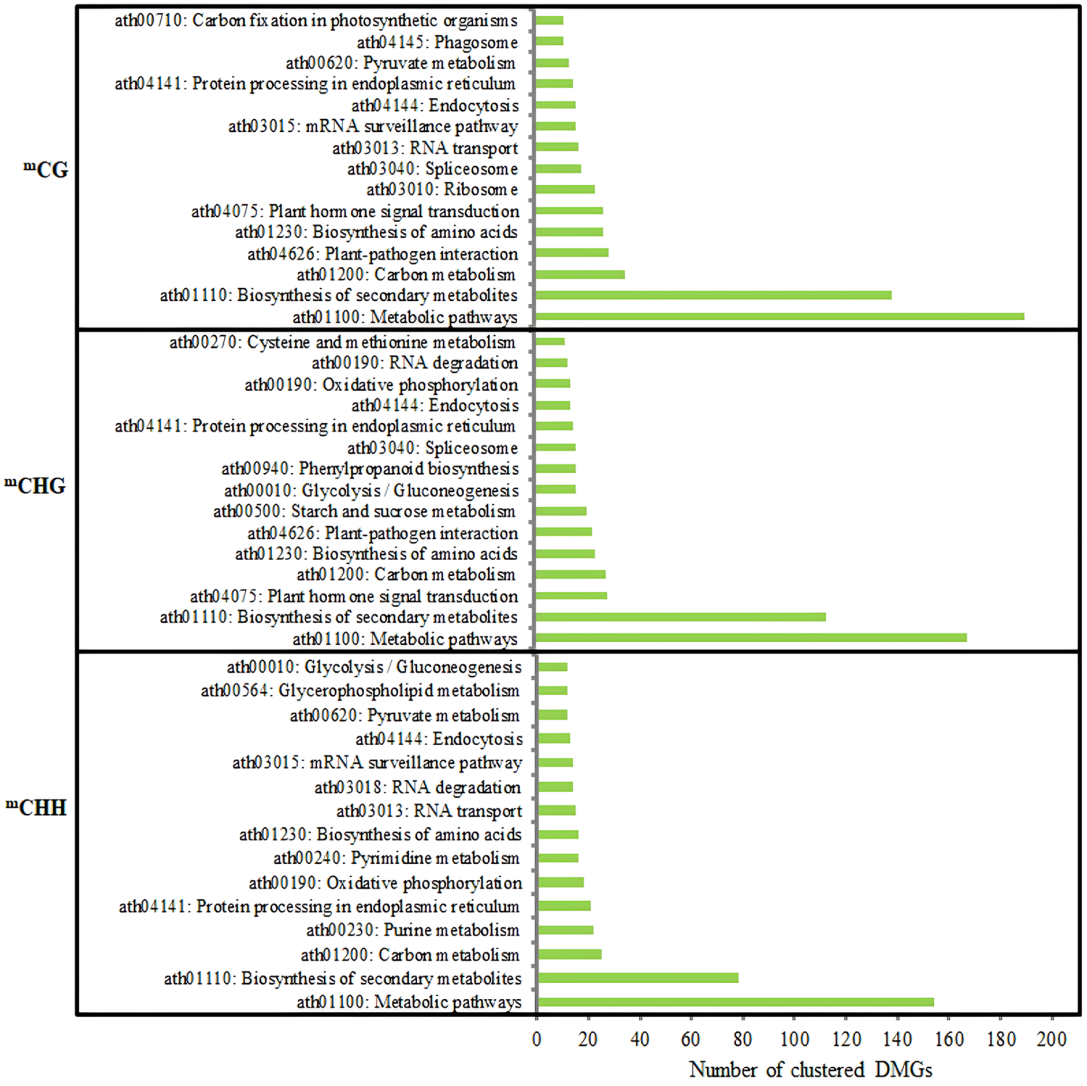
Analysis of the KEGG pathway of DMGs (top 15) in three contexts between DPs and GPs. KEGG, Kyoto Encyclopedia of Genes and Genomes; DMGs, differentially methylated genes; DPs, donor plants; GPs, grafted rubber tree plants.

KEGG pathway enrichment analysis of DMGs showed that biosynthesis of secondary metabolites and metabolic pathway were significantly enriched pathways in three methylation contexts (**Figure 11**).

### 3.7 Analysis of DMGs in a qPCR assay

**Figure 12:**
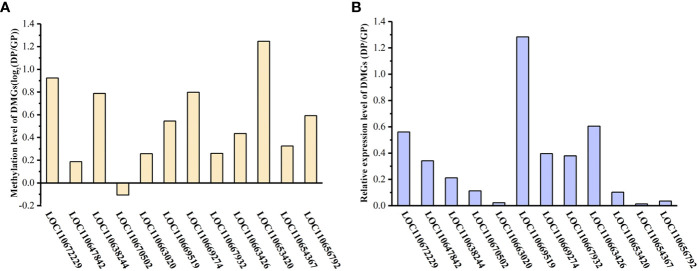
Gene expression level of DMGs. **(A)** Differences in the methylation of DMGs between DPs and GPs. **(B)** The expression level of DMGs by qPCR analysis. DMGs, differentially methylated genes; DPs, donor plants; GPs, grafted rubber tree plants.

To demonstrate that the expression of DMGs is related to DNA methylation, 12 DMGs were selected to perform the analysis of gene expression by qPCR. A total of 10 of the 12 DMGs had higher methylation levels in DPs than in GPs and showed lower gene expression in DPs than in GPs, suggesting a significantly negative relationship between gene expression and DNA methylation. However, there is a higher methylation level of DMG (LOC110670502) in GPs than in DPs, and its expression level was higher in GPs than in DPs, while there is a lower methylation level of DMG (LOC110669519) in GPs than in DPs and its expression was lower in GPs than in DPs (**Figure 12**). These data indicated that the expression of DMGs was correlated with DNA methylation.

## 4 Discussion

Grafting is a traditional agricultural propagation technology widely employed to reform crop yield, quality, and resistance to environmental stresses. Rubber tree is one of the successful commercially grafted woody plants. Previous research in *Arabidopsis* has demonstrated that grafting can modulate DNA methylation patterns within the genome of the scion cells, leading to physiological alterations (Molnar et al., 2010; Jeynes-Cupper and Catoni, 2023). Additionally, studies conducted in a Solanaceae interspecies grafting system have shown graft-induced modifications in DNA methylation (Wu et al., 2013). In woody species, graft-induced DNA methylation alterations were also reported in rubber trees and apple trees (Uthup et al., 2018; Perrin et al., 2020). Collectively, the above studies suggest that grafting can induce changes in DNA methylation, potentially affecting agronomical traits in crops.

Despite the application of MSAP analysis to investigate DNA methylation profiles in rubber tree heterografts (Uthup et al., 2018), studies regarding rubber tree whole-genome DNA methylation are not sufficient. In this study, we performed whole-genome DNA methylation analysis of GPs and DPs by WGBS. Our findings revealed that grafting triggers the reprogramming of DNA methylation, with a noteworthy decrease in genome-wide CHH methylation observed in GPs. In eggplant, grafting was able to induce grafted-plant vigor, and the enhanced plant vigor was associated with genome-wide CHH hypomethylation in the scions (Cerruti et al., 2021). Likewise, in *Arabidopsis*, a genome-wide CHH methylation decrease was also correlated with hybrid vigor (Groszmann et al., 2011; Greaves et al., 2012). Despite the highly stochastic nature of plant CHH methylation (Harris and Zemach, 2020), it is pertinent to note that DMRs were almost exclusively hypomethylated (Cerruti et al., 2021). It is possible that gene expression changes linked to enhanced vigor were associated with CHH methylation alterations. In addition, CHH methylation is inversely associated with the expression of transposable elements (TEs; Cerruti et al., 2021), which can regulate their own transcription and impact proximal gene expression through DNA methylation (Liu et al., 2022). Given that rubber trees are cross-pollinated crops and that rootstocks in grafting are typically unselected seedlings, variability in DNA methylation patterns may arise among grafted trees. Therefore, genome-wide CHH methylation decrease in GPs may be associated with intraclonal variations in the grafted rubber trees.

DNA methylation is a common epigenetic regulator of gene expression. DMR represents a notable mark of epigenetic variation and may be related to regulating the impact of DMGs on biological processes (Li et al., 2017). In total, 4,756 DMR-associated genes and 1,183 DMR-associated promoter genes were identified between GPs and DPs, respectively. Transcriptional analysis revealed that there were 9,798 DEGs in the DP and GP comparison. Furthermore, 1,698 overlapping genes were identified between DEGs and DMGs. GO annotation enrichment analysis showed that these overlapping genes were associated with various biological processes and were markedly enriched in metabolic pathway and biosynthesis of secondary metabolites. These findings suggest that graft-induced changes in DNA methylation and gene expression may affect the growth and rubber yield of the bud-grafted clones, potentially contributing to observed intraclonal variations in grafted rubber trees.

## 5 Conclusions

Whole-genome DNA methylation analyses were performed using WGBS technology to compare genomic methylation patterns between GPs and DPs. The study revealed downregulation of DNA methylation and demonstrated that grafting induced a reprogramming of DNA methylation in GPs. A total of 5,939 DMGs and 9,798 DEGs were identified between GPs and DPs, with 1,698 genes showing overlap between DEGs and DMGs. These overlapping genes were markedly enriched in the metabolic pathway and biosynthesis of secondary metabolites, as determined by KEGG pathway analysis. Global DNA methylation and transcriptional analyses further revealed a correlation between DNA methylation reprogramming and gene expression in grafted rubber trees. The study provides a whole-genome view of the methylome in rubber trees and an insight into the molecular mechanisms underlying the intraclonal variations existing in commercial planting grafted rubber trees.

## Data availability statement

The datasets presented in this study can be found in online repositories. The names of the repository/repositories and accession number(s) can be found in the article/**Supplementary Material**.

## Author contributions

H-LL: Writing – original draft, Writing – review & editing. YiW: Writing – original draft, Writing – review & editing. DG: Writing – original draft, Writing – review & editing. J-HZ: Writing – original draft, Writing – review & editing. YuW: Writing – original draft, Writing – review & editing. H-FD: Writing – original draft, Writing – review & editing. S-QP: Writing – original draft, Writing – review & editing.
