# Supplementary material for: Reprogramming of DNA methylation and changes of gene expression in grafted Hevea brasiliensis
Source: Front Plant Sci. 2024 Jun 24;15:1407700. doi: 10.3389/fpls.2024.1407700 (PMC11228250; doi:10.3389/fpls.2024.1407700)
Supplement: Supplementary file 1 [file Table_1.docx]

**Table S1. Primers used for quantitative real-time PCR**

| Primer name | Primer sequence (5’ to 3’) |
| --- | --- |
| QHbACTb7-F  QHbACTb7-R  QHb14-3-3-F  QHb14-3-3-R  QHbARF2 -F  QHbARF2 -R  QHbAUX5 -F  QHbAUX5 -R  QHbERF6 -F  QHbERF6 -R  QHbERF26-F  QHbERF26-R  QHbLBD37-F  QHbLBD37-R  QHbLBD38-F  QHbLBD38-R  QHbLBD41-F  QHbLBD41-R  QHbBTBD-F  QHbBTBD-R  QHbBTBD4-F  QHbBTBD4-R  QHbSCL1-F  QHbSCL1-R  QHbSCL23-1-F  QHbSCL23-1-R  QHbHAC1-F  QHbHAC1-R  QHbHAT1-F  QHbHAT1-R  QHbATX5-F  QHbATX5-R  QHbMT-A70-F  QHbMT-A70-R  QHbPMT2-F  QHbPMT2-R  QHbHMGR1-F  QHbHMGR1-R  QHbSRPP1-F  QHbSRPP1-R  QHbMADS12-F  QHbMADS12-R  QHbMADS-F  QHbMADS-R  QHbMADS5-F  QHbMADS5-R | CAGTGTCTGGATAGGAGGATCTA  AAATGGACCGGACTCATCATAC  TCTACCCTCCTCCACCACAG  GAGCCAGGCCAAGTCTGATT  TTGTTGGCATGGTTCGCAAG  CATCTACGCCCTCCACATCC  TCTGCTTACGACGCTTGGTT  GATAAGCCGTCCAGCTACCC  TGTAGGAGAGAGGGTCAGGC  AGTTGGTGGTTGCAGTGTGA  AACCCAGACAGTGGTGCAAA  GCAGCAAGCATCAAAACCCA  AAATCCGAACCCCCATTCCC  GCGCCGTATTTCCGGTTTAC  GTCGTGCCTTCACTGGATCA  GCGGAGATGAGGGACATGAG  CTGGCCCTGAACATCTTCGT  TCGGAGTGATCGGAGTTCCT  GTGCTGGGGATGACAGACAA  TCTCGCTTCCATCATCGCTC  CACAGAGGAGGTGCTTGGAG  GTCGCCGGACCAGGTAATAG  GAAAGATGGGACTGGACGCA  GCTCTGAGCCCTTCCAACAT  CACTGGTCTCAGGCTCCTTG  GAACGGAGATGAGAGCTCGG  ACCGAAACAAAACACCCCCT  TGCGCGAGAAGATCAACCAT  ATCTAAAAGCTGCTGGCCGT  AGAAGCCCAATGGGTAGCAC  AAGATGGAACCCGCTCTTGG  ATACCCTGCCCTTGAAGCAC  AGCCGAGAACTGAGGAGGAT  AGGTCGATGGATGAGGTCCA  TCGTTGCAGAGGACAGCATT  ACCCAATCGCATGAAGCAGA  GTTGTAAGATTCGCGTCGGC  GTTCATCCCCATTGCATCGC  TTTGGATTTTGTGCGAGCGG  AACTTGACAGCCTCAAGGGG  CTTGCTTGGAGGGAGGTCTG  TCCTCAGCAGCTGGATCTCT  TGGAAGCATGCAAGGGCTTA  AGGCTGTGTCGACTCTTTGG  GGCCCTTCCCTCAGAAAGAC  TTACGACGCTTGCAGAAGGT |
