# Supplementary material for: Reprogramming of DNA methylation and changes of gene expression in grafted Hevea brasiliensis
Source: Front Plant Sci. 2024 Jun 24;15:1407700. doi: 10.3389/fpls.2024.1407700 (PMC11228250; doi:10.3389/fpls.2024.1407700)
Supplement: Supplementary file 2 [file Table_2.docx]

**Table S2. Data summary of transcriptome sequencing for the two rubber tree samples**

| Sample | Clean Reads | Clean Bases | error_rate | Q20 | Q30 | GC_pct | total_map | unique_map |
| --- | --- | --- | --- | --- | --- | --- | --- | --- |
| DP | 45,078,466 | 6.76G | 2% | 98.24 | 94.59 | 43.53 | 42,274,976(93.78%) | 40,153,110(89.07%) |
| GP | 44,625,118 | 6.69G | 2% | 98.09 | 94.17 | 43.25 | 42,324,471(94.84%) | 40,247,435(90.19%) |
